# New Criteria for the Clinical Diagnosis of Hyperandrogenism in Polycystic Ovarian Syndrome and the Risk of Overdiagnosis

**DOI:** 10.1055/s-0039-1693530

**Published:** 2019-06

**Authors:** José Maria Soares-Jr, Marcos Felipe Silva de Sá, Edmund Chada Baracat

**Affiliations:** 1 Department of Obstetrics and Gynecology, Discipline of Gynecology, Hospital das Clínicas, Faculdade de Medicina, Universidade de São Paulo, São Paulo, SP, Brazil; 2 Faculdade de Medicina, Universidade de São Paulo, Ribeirão Preto, SP, Brazil

The excess of body hair in women is a frequent problem at the clinic that interferes with their femininity and self-esteem,^1^ and requires attention from physicians. Classically, hirsutism is the increase of terminal hair in body regions common in the male pattern. In general, hirsutism is associated with hyperandrogenism, but not in all of the cases. In 30 to 50% of the women with mild symptoms, high androgen levels are undetected.^1^

Polycystic ovarian syndrome (PCOS) is the most common cause of hirsutism and corresponds to three out of every four cases. Adrenal dysfunction (Cushing syndrome and enzymatic deficiencies) is the second cause, followed by hypothyroidism, hyperprolactinemia, and androgen-producing tumors (ovarian or adrenal). Certain drugs, mainly anabolicandrogenic steroids, can determine cutaneous hyperandrogenism.^2^ These clinical situations are part of the differential diagnosis of PCOS, since its manifestations are translated by the exacerbation of androgenic action.

Although androgen levels in the circulation may not be elevated, the appearance of hair may be an exaggerated action of androgens in effector organs (in this specific case, in the hair follicle) with other important clinical implications from the metabolic point of view. According to the literature, the major metabolic impairment factor in PCOS patients is undoubtedly the hyperandrogenism resulting from insulin resistance found in these women.^3^ Hyperandrogenism and hyperinsulinism will trigger important metabolic alterations, such as dyslipidemias, glucose intolerance, diabetes mellitus, vascular inflammatory processes, and the metabolic syndrome itself. In addition to being diagnostic criteria, excess androgens are an important part of the disease pathophysiology and should be tackled in the treatment of patients. Thus, androgen dosages and the search for signs of hyperandrogenism are essential in approaching patients with suspected PCOS.^4^ When evaluating hyperandrogenism in women with suspected PCOS, some considerations must be made in the light of new concepts about the disease.

The conventional dosage of circulating levels of testosterone is quite inaccurate the female gender,^4^ and sometimes does not reveal the hyperandrogenism that determines less intense forms of hirsutism. In fact, a quarter of hirsute women with PCOS do not have the corresponding hyperandrogenism (testosterone, androstenedione, and dehydroepiandrosterone sulfate), which may raise doubts about the diagnosis. In general, this is determined by biochemical tests employing radioimmunoassay or electrochemiluminometric methods, in which the calibration curve of the kit is the same as that developed for the male sex. In part, this may explain the noncorrelation between serum levels and the level of hirsutism. There are more sensitive methods, such as liquid or gas chromatography coupled with mass spectrometry, but they are unfeasible in the clinical practice, given their high costs. Because of this difficulty in the laboratory diagnosis of PCOS, the clinical evaluation of hyperandrogenism through its more frequently observed manifestations, such as acne and hirsutism, becomes relevant.^2^ ^3^ ^4^ Therefore, the evaluation of hirsutism is important for the clinical diagnosis and for the initiation of treatment.

In 2018, Teede et al^5^ organized a major and careful review of the recommendations for the diagnosis and treatment of women with PCOS. Acne, alopecia, and hirsutism are markers of cutaneous hyperandrogenism in this guideline. Researchers also suggested the Ferriman-Gallwey (F-G) index as a tool for the diagnosis and follow-up of women with PCOS and hirsutism. However, the great doubt of some researchers is the cutoff point of this index for these recommendations,^6^ ^7^ ^8^ ^9^ ^10^ because ethnicity has great relevance in determining hair distribution in women. The most intense hirsutism can be observed in women of the Middle East, of Latin America, and of the Mediterranean.^5^ Since milder degrees can be considered as normal patterns for this ethnic group,^6^ ^7^ ^8^ ^9^ ^10^ they are not a reason for seeking medical care. This fact raises discussion about the therapeutic decision of the clinician by keeping in mind the side effects of antiandrogen therapies.^6^ ^7^ ^8^ ^9^ ^10^

Classically, most of the medical literature and of the consensuses considered hirsutism when the F-G index was ≥ 8.^6^ ^7^ ^8^ ^9^ The recommendations by Teede et al^5^ have lower cutoff values, namely: six for Caucasian and Afrodescendant women, and four for women of Oriental/Asian origin, such as Chinese, Japanese, Korean, and others. During the evaluation by the physician, this may cause some confusion, since hirsutism can be diagnosed in many women without any complaint or other concomitant symptoms. Psychologically, the diagnosis of hirsutism carries an emotional load for women, which can lead to stigma, especially in adolescents.^5^ Thus, the questioning about the need to apply the F-G criteria without the woman's complaint.

Another concern would be prescribing treatment for an alleged hyperandrogenism based on a diagnosis of hirsutism that would not actually exist. Antiandrogenic drugs are used for this purpose and can have important side effects, ranging from decreased libido to feminization of the male fetus. Therefore, additional care and measures are necessary, such as the associated prescription with contraceptive methods.^11^ Some situations can still be confused with cutaneous hyperandrogenism, such as hypertrichosis, which is the increased pilification in regions specific for women and growth of lanugo (unpigmented hair) on the shoulders and on the forehead caused by some drugs (progestogens, glucocorticoids, cyclosporins, diazoxide, valpronates, minoxidil, penicillamine), or by diseases not associated with hyperandrogenism (hypothyroidism, anorexia, porphyria, dermatomyositis) that may even be constitutional.^11^ Thus, the importance of using the F-G index carefully and identifying other nonspecific manifestations of hyperandrogenism not only for the diagnosis, but also to avoid inappropriate prescription of antiandrogenic drugs.

Finally, hirsutism is a frequent complaint during gynecological consultations, but the interpretation of the physical examination, as well as the application of the recommendations by Teede et al,^5^ demand care when this symptom is isolated and does not bother the woman. There is a phenotype of women with PCOS who ovulate and present sonographic images of polycystic ovaries, but may have mild hirsutism.^12^ This is concerning, as these women can ovulate and become pregnant more often than others with phenotypes of menstrual dysfunction. Another point would be whether mild hirsutism should be mentioned to the woman: would we be creating a stigma that did not exist before the consultation? There is insufficient data in the literature for answering this concern. Therefore, the wisdom of each professional should prevail and perhaps new studies may review the cutoff points of the F-G index in the clinical practice in order to avoid the risk of overdiagnosis.
